# Content-Based Discovery for Web Map Service using Support Vector Machine and User Relevance Feedback

**DOI:** 10.1371/journal.pone.0166098

**Published:** 2016-11-18

**Authors:** Kai Hu, Zhipeng Gui, Xiaoqiang Cheng, Kunlun Qi, Jie Zheng, Lan You, Huayi Wu

**Affiliations:** 1 State Key Laboratory of Information Engineering in Surveying, Mapping and Remote Sensing, Wuhan University, Wuhan, China; 2 School of Remote Sensing and Information Engineering, Wuhan University, Wuhan, China; 3 Faculty of Resources and Environmental Science, Hubei University, Wuhan, China; 4 National Engineering Research Center for Geographic Information System, China, University of Geosciences, Wuhan, China; 5 Faculty of Computer Science and Information Engineering, Hubei University, Wuhan, China; 6 Collaborative Innovation Center of Geospatial Technology, Wuhan University, Wuhan, China; University of Illinois-Chicago, UNITED STATES

## Abstract

Many discovery methods for geographic information services have been proposed. There are approaches for finding and matching geographic information services, methods for constructing geographic information service classification schemes, and automatic geographic information discovery. Overall, the efficiency of the geographic information discovery keeps improving., There are however, still two problems in Web Map Service (WMS) discovery that must be solved. Mismatches between the graphic contents of a WMS and the semantic descriptions in the metadata make discovery difficult for human users. End-users and computers comprehend WMSs differently creating semantic gaps in human-computer interactions. To address these problems, we propose an improved query process for WMSs based on the graphic contents of WMS layers, combining Support Vector Machine (SVM) and user relevance feedback. Our experiments demonstrate that the proposed method can improve the accuracy and efficiency of WMS discovery.

## Introduction

The number of online GIServices has increased dramatically in recent years with the development of geospatial sharing technologies and the emergence of many open government data initiatives. According to a recent global-scale resource survey of the Web Map Service (WMS) standard proposed by Open Geospatial Consortium (OGC), there are more than 40 thousand valid WMSs publicly available globally on diverse map subjects, geographic extents and projections [1]. The booming of online GIServices has enhanced geospatial research and applications, but also has created new challenges for GIService discovery and selection.

To improve discovery of desired geographic information services (GIServices) researchers have proposed different methods to improve web service metadata, data provenance, and query methods. Subject-specific web crawler techniques have been widely researched as a means to improve the effectiveness and efficiency of GIService discovery [1,2], but manual searches for a desired service among a huge amount of irrelevant and unqualified services is still a painful process, a number of solutions have been proposed. Semantic searching methods can increase search precision and recall [3] by constructing a semantic reasoning relationship network of different GIServices prior to conducting search. For example, a search term including a keyword “water” yields GIServices for seas and rivers, surpassing the results found by keyword matching by matching meanings. A performance monitoring mechanism also provides a criterion to filter unqualified GIServices and locates desired GIServices by dynamically collecting quality information for the corresponding services [4]. Appropriate client-side visualization methods and human-computer interaction mechanisms can refine query criteria, narrow the search range and the enrich user experience in GIService selection [5–7], e.g., Microsoft PivotViewer, quality view and layer thumbnail view. To improve the service locating efficiency, GeoSearch combines semantic search, quality of service and data visualization together into an integrated search brokering framework [7]. Even when these methods are applied however, two issues remain:

1. The problem of mismatching between GIService graphic content and GIService semantic description is an ongoing issue. Most GIServices can be presented with image forms that cannot be properly described with text-based semantic descriptions. For example, there are GIServices with complete metadata information on service abstracts, keywords and even response time, but invalid graphic content. In other cases, a GIService might be named with a name of administrative region but actually presents thematic maps like point of interest (POI) mapping or building maps.
2. The problem of semantic gaps in image-based GIService discovery occurs because of the difficulties in describing the meaning of graphic content. For example, people can identify the land use type by checking the graphic content of a land use thematic mapping service, but computers however, can only tell the RGB values, not the high-level semantics of the graphic content.

To deal with the first problem, we propose the use of image features to search WMSs. Nowadays, though there is little research on the quality of WMS content, much research has been conducted in the fields of Image Quality Assessment [8] and Map Quality Assessment [9]. In terms of Image Quality Assessment, the metrics such as lightness, luminance, contrast, and clarity are proposed as the measurements to build up an assessment model [10]. The Scale-Invariant Feature Transform (SIFT) is also used to assess the quality of photo graphics [11]. However, these methods do not fit WMS scenarios.

Visualizations of WMS layers are a multi-dimensional expression of map data, the rendering result is based on the map geometries (i.e., point, polyline and polygon) and their relations. Image features like lightness and contrast can only express the characteristics of rendering styles and cannot sufficiently express the characteristics of a WMS. In another aspect, quality in Map Quality Assessment research is expressed through indexes like map readability or map information volume. Map readability focuses on the name placement problem [12] while map information volume offers quantitative solutions to estimate the map quality [13] from the perspective of information quantity or content conveyed by a map. However these methods cannot sufficiently express the quality of a WMS. The architecture of layer combinations in WMS allows every map layer to only express partial characteristics, a Point of Interest (POI) layer expresses spatial point characteristics, road layer represents the spatial polyline characteristics or land use layer represents a spatial polygon characteristic. The description quality of image features contributes to search accuracy. So, it is important to designate the corresponding image features for WMS layers to improve the efficiency of content-based WMS discovery.

To deal with the second problem, we need to understand that the quality of discovery results depends on fulfilling the user expectations. Both human users and computers need semantics to make a judgment based on the graphic content. Unlike computers, human users tend to understand high-level semantic, unstructured graphic content, and thus there is a semantic gap between human and computer understanding [14]. Research on Content-based Image Retrieval (CBIR) shows that semantic gaps can be narrowed by using “searching with an example” or by incorporating user feedback in an image retrieval application [15]. Searching with an example means that users find an example of their desired image and by using certain image feature computation of similarities to the example, the computer acquires candidate images and users select among the returned images[16]. User relevance feedback is a supplementary method for image retrieval [17], because a search algorithm always has shortcomings and might give wrong results. Users provide feedback by specifying right or wrong images to improve search results in the next retrieval cycle. These methods can improve the accuracy of WMS discovery.

Based on these understandings, we propose a content-based method to search WMS layers by building up WMS layer specified image features, searching with examples, and an added user feedback mechanism. In particular, the WMS layer specified image features describe the graphic content both spatially and graphically. Searching with examples uses the Supported Vector Machine (SVM) to combine knowledge of positive or negative images. The user feedback uses the mouse-tracking method to collect the user feedback with low user burden so users do not need extra energy to answer designed questions. Finally, through experiments the proposed method in this paper is demonstrated to be efficient.

The rest of the paper is organized as follows: section 2 introduces the workflows of a WMS layer search, the design of a WMS layer specified image feature, the user feedback mechanism with front-end script library, and the classification process for WMS layers. Section 3 discusses WMS layer search experiments. Section 4 discusses the advantages and disadvantages of the proposed approach based on the experiment results. Section 5 draws some conclusions.

## Methodology

### 2.1 WMS layer searching workflow

**Fig 1:**
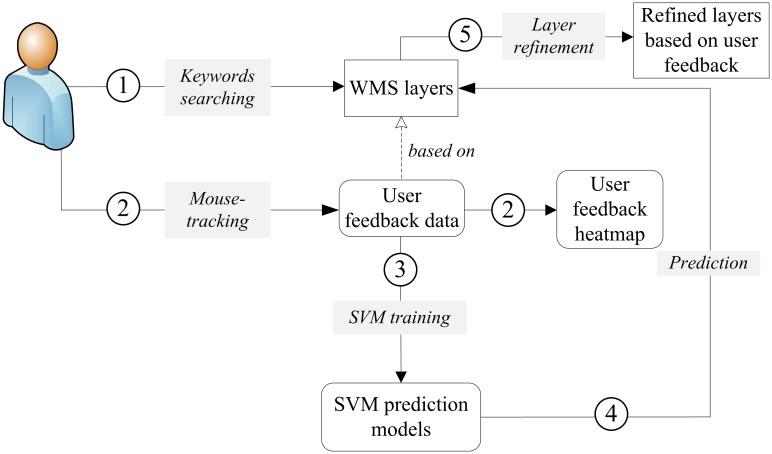
WMS layer discovery workflow combining image features and user feedback.

Traditional search methods for WMS ask users to specify the geographic extent, map subject, provider name and other query constraints described in keywords. Users need to check the returned WMS layers one by one manually. In this paper, we extend existing search methods with a search workflow that combines image features and a user feedback mechanism. Fig 1 presents the proposed search workflow.

As in traditional discovery processes, users also need to specify keyword-based query constraints in this workflow. The results appear as WMS layers with thumbnails shown in the Graphic User Interfaces (GUIs). Users can evaluate the resulting WMS layers visually through interactive devices. In our case, the mouse is the interactive device and collects user feedback data such as clicking actions and the time a user lingers over a part of the GUIs. We assume that lingering over parts of the interface reflects user interest in these areas. This user feedback data is used to tag WMS layer candidates during SVM training. The generated SVM prediction models predict, refine, and get the final results. In this way, the proposed search workflow can improve the accuracy and precision of WMS discovery processes, and therefore the user experience.

This proposed implementation is similar to the “searching with an example method” in CBIR research. However, searching with an example can also create user burdens so users must prepare the example prior to a search. In our case, we use the search results from a keyword-based search as the input examples. The example input into the search engine is not just one example but a collection of examples with tags on interesting and not interesting areas to reduce the user burden.

Keyword-based searches are an important step of the workflow but not the focus in this paper. Instead, we discuss mechanisms for combining user feedback data and the SVM classification process. To implement this search functionality, an image feature is built based on the characteristics of the WMS layer content. These image features can be used as mathematical vectors in computing similarities between images or for classification purposes. In addition to features used in the similarity or classification computing, the mechanism for collecting user feedback is also important. The challenge in user feedback lies in collecting the feedback data authentically and using them properly in building the prediction models. In the next sections, the methods used to construct image features, acquire user feedback data, and build the prediction models are detailed.

### 2.2 WMS layer specified image feature construction

**Fig 2:**
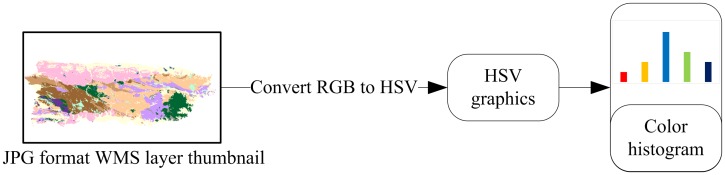
Color histogram extraction process.

To build up an image feature to better express the unique characteristics of individual WMS layers, we selected the HSV color model since it is better for user cognition than RGB model [18]. Additionally, the graphic representation of WMS layers are seen by users and provide the basis for collecting user feedback, therefore HSV is a better choice in our WMS layer search scenarios. We applied color histogram matching methods as they are commonly used methods in CBIR research. Our color histogram matching method is illustrated in Fig 2.

**Fig 3:**
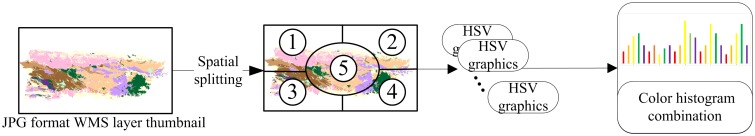
Image feature construction of a WMS layer based on spatial splitting.

The extraction process is simple and easy to conduct. However, a shortcoming is that color histogram matching does not account for the spatial distribution of the graphic content in a WMS. Color pixels in different regions might be wrongly regarded as the same content during the feature extraction process. To consider the spatial distribution of color pixels, we adopted a method similar with Spatial Pyramid Matching (SPM)[19]. SPM splits the candidate images into pyramid tiles and calculates the numerical values representing the frequency of discrete colors to create a color histogram, as shown in Fig 3. Then these values are stored in the order of the tiles and are used to compute the similarity between examples and candidates. In this way, the image matching based on similarities considers both color and spatial distribution. The same color in different regions will not be regarded as similar, thus avoiding mismatches.

During spatial splitting, as seen in Fig 3, the ellipse and four cardinal lines split the graphic content of the WMS layer. For computational purposes, we assigned the long axis of the eclipse as the half of the image width and short axis of eclipse as the half of the image height. After splitting, the image is divided into five parts. For each of the five parts, we conducted HSV extraction. HSV values are stored as one column vector. HSV has three dimensions, H (hue) S (saturation) and V (value). To make the image feature more capable of expressing the image characteristics of a WMS, we set the parameters of the regions to 3, 8, and 12, for H [0,179], S [0,255], and V [0,255], respectively. After splitting, every WMS layer thumbnail is used to generate a column vector with (3+8+12)*5 = 115 elements. These vectors are the foundation for computing similarity between WMS layers.

### 2.3 User feedback data acquisition based on a front-end script library

We used the mouse-tracking method to collect the user relevance feedback when user viewing the thumbnail image of the result WMS layer. Many methods have been proposed to acquire the user feedback including eye-tracking [20], body-tracking, and mouse-tracking devices [20]. Eye-tracking and body-tracking have the advantages of being a natural way to collect body reactions of users. Users do not need to do any extra work to offer the feedback, thus the user burden is low providing implicit feedback. However, eye-tracking and body-tracking methods often require expensive devices. In contrast, mouse-tracking is the most often used feedback mechanism as feedback can be collected from personal computers with a connected mouse or a touch pad. This makes this approach applicable in many scenarios requiring use feedback.

**Fig 4:**
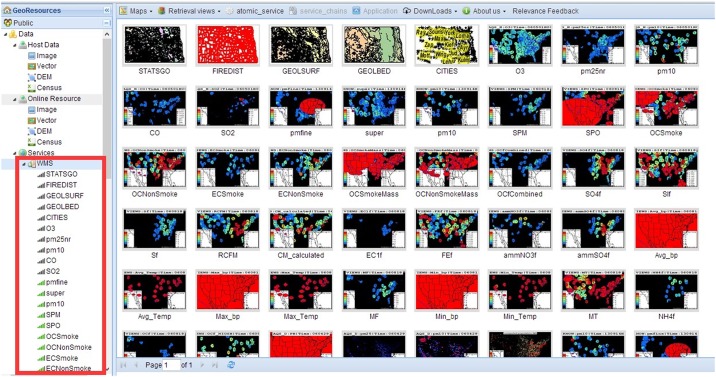
WMS layer thumbnails.

We designed a prototype of web-based application that permits users to volunteer their feedback in the interactive area of the web page. It is implemented upon our previous system, GeoSquare, a collaborative online sharing and geoprocessing platform [21,22]. The JavaScript library Heatmap.js was used to implement the user feedback collection mechanism. The web application exploits HTML elements in the GUIs as mapping canvases and collects mouse actions by recording clicks and movements. The coordinates of the mouse pointer are recorded as tracking points. These coordinates in turn highlight out objects of interest. In this case, the objects are the WMS layer thumbnails, as illustrated in Fig 4.

As Fig 4 shows, the text appearing under these images are the titles of the corresponding layers. We can tell that most of the layer titles do not adequately explain the content of the images because too many abbreviations are used and might be confusing to users. In this case, a content-based WMS search is appropriate and necessary.

**Fig 5:**
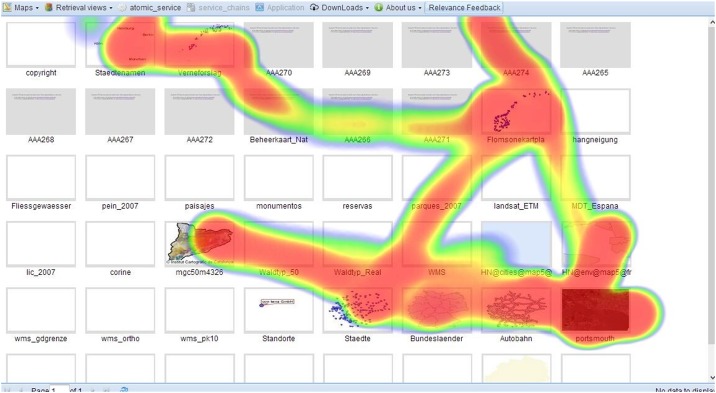
User feedback heatmap after user interaction.

The GUI illustrated in Fig 4 displays areas where users interact with WMS layer thumbnails. Since mouse point coordinates are recorded, and assuming that users prefer a WMS layer thumbnail if they linger over it longer or click it to visualize the enlarged image, then point counts imply areas of interest. In our method, the user burden when collecting user feedback is minimized because users do not feel the point collection process. A user feedback heat map is shown in Fig 5, showing the tracking pattern over a series of WMS thumbnails.

As illustrated in Fig 5, the red parts in the heat map represent the areas that users spend more time on, and the yellow parts (around the layer named “AAA266” in second row of thumbnails) are the areas where users spend less time. In this figure, we can see several WMS layers are blank or grey with no graphic content. These layers are invalid WMS layers, however they still provide title information, abstracts, or keywords. So it is important to search the WMS layers considering the level of graphic content.

### 2.4 WMS image classification based on SVM

A Support Vector Machine (SVM) is defined as follows: given a linear separable samples of (xi, yi), then i = 1, 2…N, y_i_ ∈ (−1, 1), x_i_ ∈ R_d_. Assume that a hyperplane w ∙ x + b = 0 can separate the positive and negative data, then the plane is the classification hyperplane. The classification of the corresponding function is f(x) = w∙x + b. In f(x), solving the optimal plane after normalization, is equivalent to minimizing ∥w∥, and the objective function can be written as:

$$\text{min}ø\left(\text{w}\right)=0.5\cdot ∥\text{w}{∥}^{2}$$ (1)

The constraint conditions of the formula is y_i_(w ∙ x + b)– 1 ≥ 0, i = 1, 2, …N. Define N as Lagrangian function a_*i*_, i = 1…N; then, by solving this quadratic optimization problem, we get the optimal classification plane, including $\text{w }=\;\sum_{\text{i}}^{\text{n}}{\text{a}}_{\text{i}}{\text{y}}_{\text{i}}{\text{x}}_{\text{i}}$, where *x*_*i*_ is the sample located in the classification surface, called the support vector. The classification function is written as:

$$\text{F}\left(\text{x}\right)=\text{sign}\left(\sum_{i=0}^{N}a_{i}y_{i}x_{i}\cdot \text{x }+\text{ b}\right)$$ (2)

This nonlinear problem can be solved by mapping the input space to a high-dimensional space through the kernel function K(x, y). The classification function can be converted to:

$$\text{F}\left(\text{x}\right)=\text{ sign}\left(\sum_{i=0}^{N}a_{i}y_{i}x_{i}\cdot \text{K}\left(x_{i},\text{ x}\right)\;+\text{ b}\right)$$ (3)

Through transformation, this function is used to calculate the distance between the image characteristics of a WMS and the classification hyperplane, the distance can be used to describe the similarity between a target image and retrieved images. The classification function can be transformed into:

$$\text{F}\left(\text{x}\right)=\;\sum_{i=0}^{N}a_{i}y_{i}x_{i}\cdot \text{K}\left(x_{i},\text{ x}\right)\;+\text{b}$$ (4)

A requirement of user feedback is that users need identify irrelevant data to improve the accuracy in the next query. In the case of Fig 4, a total of 35 WMS layer thumbnails were shown in the GUI, users are interested in seven layer thumbnails, and the remaining 28 layer thumbnails were not interesting to users. The interesting WMS layers were marked as 1; the uninteresting WMS layers are marked as -1. According to our proposed image feature construction process, we can calculate the vector values for graphical map layer features to facilitate SVM classification. By means of positive data marked as 1, including seven WMS layers, and negative data marked as -1 with 28 WMS layers, we trained our SVM prediction model. The model was used to compute the possibility of a layer belonging to the class of images interesting to users.

## Experiments and Results

We designed two groups of experiments to verify whether the proposed user feedback mechanism and the image features can be used to capture and understand the human semantics. In these experiments, the web application collected the image features from map layer thumbnails in areas of interest through mouse-tracking to identify interesting layers and conducted layer filtering. We used a WMS layer dataset collected in advance as test dataset, a total of 653 WMSs with 11689 available layers. The thumbnails in JPG format of map layers were converted into separable linear samples to calculate image features and similarity. The separable linear sample for SVM training is the 115 * 1 column collection. We used the LibSVM library[23] to compute the prediction models and regarded as a hyperplane for classification. Every candidate WMS layer thumbnail is calculated as a feature vector. The distance to the hyperplane measures the similarity. A positive distance value means that this WMS layer belongs to the interesting group and a negative distance value places this WMS layer in the uninteresting group. The absolute value of the distance measures image similarity to the interesting or uninteresting group.

**Fig 6:**
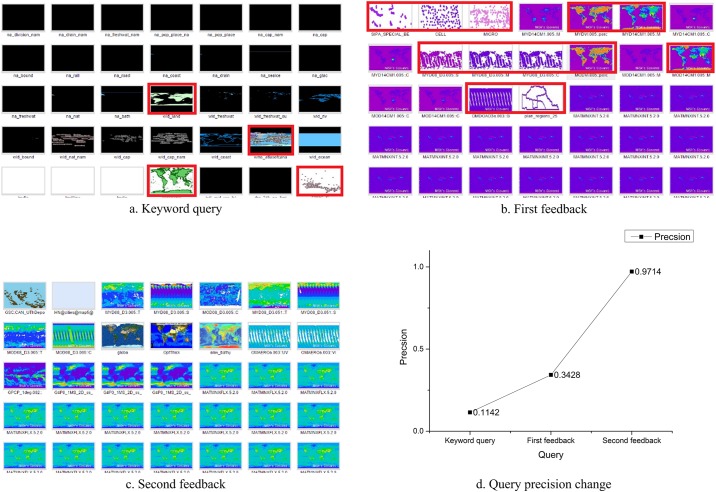
WMS layer query experiment for retrieving layers with abundant map information.

**Fig 7:**
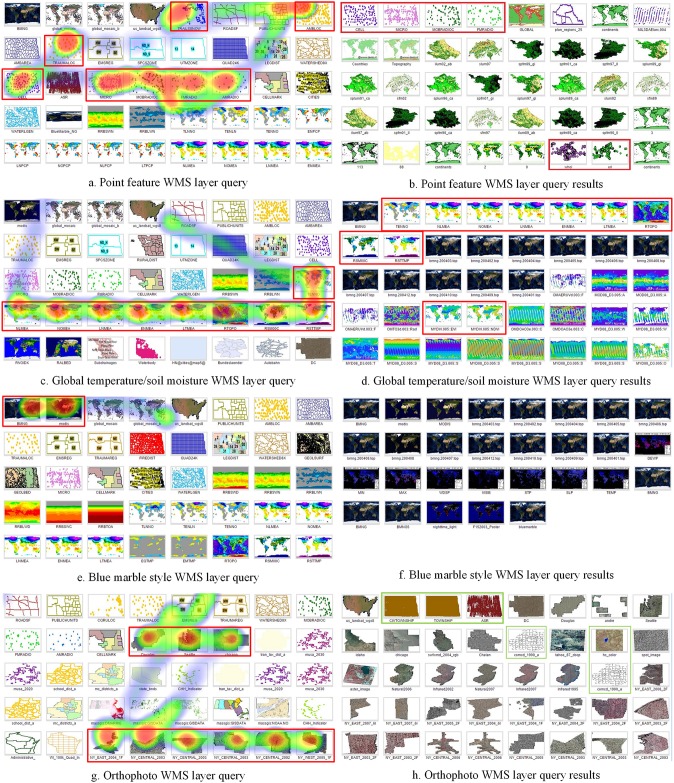
WMS thematic layer query experiment results.

In the first group of experiments, we aimed at verifying whether the proposed user feedback mechanism can effectively utilize image features to distinguish the WMS layers with abundant map information from the blank layers. We asked users to specify WMS layers that have relatively abundant map content. We recorded the experimental results in three search scenarios: only keyword queries, first user feedback, and second user feedback queries. In the keyword queries, the sample and fundamental query were used to assign the spatial range of user specific regions. For example, users assigned the desired geographic range as longitude and latitude coordinates and designated the service type as WMS. The first and second feedback used the same refinements in the keywords but combined a keyword search with user feedback information. By combining a keyword search with the user feedback information, the query results filtered out thumbnails that were uninteresting to users. The first feedback results were the filtered results based on the keyword query and the second feedback results were based on the first feedback results. The three query results are shown in Figs 6a, 6b and 7c. Fig 6d shows the precision improvement after combining user feedback.

In the keywords search results, keyword queries only filter WMS layers by comparing metadata information. The query results were not complete or irrelevant as only four of 35 WMS layers presented rich WMS layer information in a keyword query. In the first user feedback results, after acquiring user-identified interesting regions, the number of interesting WMS layers increased to 12 out of 35 layers. In the second feedback results, the uninteresting WMS layers were reduced to one layer (the one in the first row and the second column of Fig 6c, which had little information and selected by mistake). Despite this error, user preferences are reflected in our user feedback mechanism and the overall WMS layer query precision increased.

In the second group of experiments, we aimed to verify the capability of the image features in capturing and understanding the user semantics of different map feature types. Four query scenarios were designed using the user feedback mechanism for searching thematic map types including point feature maps, global temperature/soil moisture maps, blue marble style images, and orthophotos. As illustrated in Fig 7a, eight (20%) WMS layers that contain point features were specified as user expectations in the second query using user feedback mechanism from the 40 candidate layers retrieved in previous query. But, Fig 7b shows that only six (15%) point feature maps were correctly retrieved. The other 34 unmatched layers were wrongly selected as they had similar color feature to the sample point feature layers, as they were different map feature types, e.g., land use maps. Similarly, as seen in Fig 7c and 7d, nine (22.5%) WMS layers related to global temperature or soil moisture were identified as query constraints, however only 11 (27.5%) of the layers in the results list were correctly retrieved. Query precision was high for many particular thematic map types, as illustrated in Fig 7e and 7f, although only two (5%) blue marble styled WMS layers were manually identified, all 29 (100%) retrieved WMS layers were correct. In Fig 7g and 7h, according to the nine (22.5%) selected orthophoto maps, the user feedback query retrieved 34 (85%) orthophotos and only six (15%) WMS layers were wrongly selected.

## Discussion

The two groups of experiments presented in section 3 provide a basis for understanding the reasons for variations in the query precision related to the type of query. These experimental results also expose the advantages and disadvantages of our proposed method. In the first group of experiments, we selected keywords as the query constraints for searching WMS layers, the thumbnail results showed that many of the WMS layers were not efficient or invalid. This illustrates that many WMSs are not maintained and updated. The first feedback scenario showed an increase in WMSs with abundant map information as a graphic content level comparison was executed based on input training data. These training data were generated from the way users interacted with the search interfaces. The comparison was transformed in our workflow from a subjective human-based selecting process to a matrix computation problem by converting images to image features. In this way, the semantic gaps were narrowed by linking human behaviors and computers to better understand and interpret the graphic contents of WMSs. As the training data were partially collected, it is inevitable that errors will occur in the classification process.

Our first results in the first feedback search of the first group were not as accurate as we would like. In the second feedback search, the quantity of acceptable results increased to a relatively high level and the search accurately identified the WMS layers of interesting to users because the training data in the second feedback scenario was improved by the first feedback inputs. The results show that our proposed image feature is approach an effective means to identify the abundant-information maps. The increase in the accuracy of search results depends on how the well image feature can describe WMS layer content.

In the second group of experiments, we verified the performance of the proposed WMS layers specific image feature for capturing and understanding the user semantics of different map feature types. Four types of one-time user feedback searches for thematic WMS layers including point feature maps, global temperature/soil moisture images, blue marble style maps, and orthophotos were compared. The first two queries show that our proposed WMS layers specific image feature can partially understand human semantics but can easily misled by similar visual features, Fig 7a, 7b, 7c and 7d. The query results for the point feature WMS layers are unsatisfactory. This is mainly because that the proposed image feature mechanism is not so discriminating with point feature maps. Many land-use WMS layers have color distributions very similar to the point feature layers. It is difficult for computers to distinguish the differences. The global temperature/soil moisture WMS layer results were also not accurate because thematic layers such as water vapor layers have similar color distribution and geographical layout.

The last two queries in the second group of experiment shown in Fig 7e, 7f, 7g and 7h, delivered better results. The blue marble style WMS layer results indicate that the using proposed WMS layer-specific image feature is an efficient way to identify visualization-related characteristics understandable in human terms. Similarly, the orthophoto WMS layers query achieved high precision because the orthophoto WMS layers have relatively unique color features. In general, the proposed feature performs well when searching some thematic map layers with very unique and clear image feature representation. But it performs not so well for those feature types that are not unique enough or have mixed and complex semantic meanings. In addition, user feedback may provide insufficient or incorrect information.

End-users may often conduct unexpected behaviors, such as hovering over uninteresting areas for long times that will mislead the computer in interest feature extraction. Under fitting or over fitting in the SVM learning process may also affect the classification. This can easily happen when end-users have multiple interesting targets and select thematic map layers containing more than one visual or thematic type, or when a user provides insufficient map samples. Multiple image features can occur in one map, thus only partially considering certain features in the feedback can affect the results. For example, many WMS layers may have similar spatial distribution but different color proportions. If only partial characteristics are considered in the user feedback, color proportion or spatial distributions, the characteristics captured will be not clear and the precision of the search will be affected.

In this paper, the proposed WMS layers specific image feature construction approach was not compared to other image feature construction methods, such as the SIFT features or other mixed features. Our future work will describe the WMS layer graphic content with different features and performance measures. In particular we will consider the graphic contents of WMS layers, and will introduce image features of typical layer geometry into search and service discovery. While image features were critical to successful final search results, they are not the only elements in a successful search. Communication between human and machines is similar to human to human communications. The results are the process of trial and error with many adjustments. A system that learns from user feedback can make search and discovery more intelligent and helpful for users.

User feedback is universal in every human-computer interaction application scenario. However, the user burden is an important issue to tackle. We are often confronted with situations that are not natural and participation in the user feedback collection such as answering questionnaires or filling in electronic tables. If these feedback tasks are not relevant to user needs they might be a waste of time from a user perspective. If the user burden takes too much energy, the quality of the resulting data can be affected. In this paper, user feedback is gathered unobtrusively by tracking mouse movements in web GUIs using a client-side library. User burden was efficiently reduced.

Compared with traditional service searches, both image features and user feedback are used to improve the search process and search results. Image features are not traditionally used for WMS searches because map images involve multiple representations and CBIR technology cannot be directly applied. User feedback is often discussed in the context of their usability or fitness for use in GIService applications and studied in terms mapping content with eye-tracking equipment and seldom discussed in relation to applications using the feedback as the computational parameters in the real-time searches. Our proposed methodology fills this gap and represents a means for applying new IT technologies in the GIService field. In addition to OGC-standard compliant GIServices for searching online mapping service in ordinary applications, our approach might be meaningful and potentially valuable in commercial applications.

## Conclusion

To deal with the two issues of mismatching between graphic content and semantic description and the semantic gap problem between user and machine understanding of the graphic content in GIServices, this paper puts forward a method for combining SVM and user relevance feedback to retrieve interesting WMSs. User feedback narrows the semantic gap and implicit feedback mechanisms, such as mouse-tracking, can effectively reduce the user burden in feedback data collection. Our experiments demonstrate that the proposed method can improve the accuracy of WMS search but the performance varies when dealing with different feature types. The proposed method can be extended to include any GIServices that can be visualized as thumbnails, such as Web Coverage Services (WCSs) and Web Feature Services (WFSs).

The proposed WMS layers specific image feature very effectively distinguishes maps with abundant information from the blank maps. It performs relatively well when searching the thematic map layers that have unique and clear feature representation, e.g., blue marble images, orthophotos and land use images. Some problems still exist however, as it performs not so well with point feature maps and maps with mixed features. Therefore, feature extraction based on color and spatial partitioning still needs improvement for effective search and identification among all types of thematic maps as we adopted the HSV divisions (8, 12, 3) from empirical values rather than quantitative indicators. Search efficiency can be improved by establishing typical area shape feature libraries to intelligently and dynamically classify WMS layers. In addition, we did not consider keyword queries; extending our proposed method with semantic web technologies could also improve efficiency and accuracy of GIService discovery. Content-based WMS layer discovery could be combined with more heterogeneous data sources such as metadata and tagging information for better search and discovery results. Future work will focus on combining semantic technology and machine learning methods to make GISerivce discovery more intelligent and efficient; querying semantic relationships among the GIServices, understanding the relationship between geographical expressions of location and human natural language, as well as relational queries in graph-structure big data.
